# The Impact of First-Time SARS-CoV-2 Infection on Human Anelloviruses

**DOI:** 10.3390/v16010099

**Published:** 2024-01-09

**Authors:** Anne L. Timmerman, Lisanne Commandeur, Martin Deijs, Maarten G. J. M. Burggraaff, A. H. Ayesha Lavell, Karlijn van der Straten, Khadija Tejjani, Jacqueline van Rijswijk, Marit J. van Gils, Jonne J. Sikkens, Marije K. Bomers, Lia van der Hoek

**Affiliations:** 1 Laboratory of Experimental Virology, Department of Medical Microbiology and Infection Prevention, Amsterdam UMC, University of Amsterdam, Meibergdreef 9, 1105 AZ Amsterdam, The Netherlands; a.l.timmerman@amsterdamumc.nl (A.L.T.); commandeurlisanne@gmail.com (L.C.); m.deijs@amsterdamumc.nl (M.D.); m.g.j.m.burggraaff@amsterdamumc.nl (M.G.J.M.B.); k.tejjani@amsterdamumc.nl (K.T.); j.vanrijswijk1@amsterdamumc.nl (J.v.R.); m.j.vangils@amsterdamumc.nl (M.J.v.G.); 2 Amsterdam Institute for Infection and Immunity, Meibergdreef 9, 1105 AZ Amsterdam, The Netherlandsj.sikkens@amsterdamumc.nl (J.J.S.); m.bomers@amsterdamumc.nl (M.K.B.); 3 Department of Internal Medicine, Amsterdam UMC, Vrije Universiteit Amsterdam, De Boelelaan 1117, 1081 HV Amsterdam, The Netherlands; 4 Department of Internal Medicine, Amsterdam UMC, University of Amsterdam, Meibergdreef 9, 1105 AZ Amsterdam, The Netherlands

**Keywords:** TTV, TTMV, TTMDV, anellome, SARS-CoV-2, virome

## Abstract

Members of the *Anelloviridae* family dominate the blood virome, emerging early in life. The anellome, representing the variety of anelloviruses within an individual, stabilizes by adulthood. Despite their supposedly commensal nature, elevated anellovirus concentrations under immunosuppressive treatment indicate an equilibrium controlled by immunity. Here, we investigated whether anelloviruses are sensitive to the immune activation that accompanies a secondary infection. As a model, we investigated 19 health care workers (HCWs) with initial SARS-CoV-2 infection, with blood sampling performed pre and post infection every 4 weeks in a 3-month-follow-up during the early 2020 COVID-19 pandemic. A concurrently followed control group (*n* = 27) remained SARS-CoV-2-negative. Serum anellovirus loads were measured using qPCR. A significant decrease in anellovirus load was found in the first weeks after SARS-CoV-2 infection, whereas anellovirus concentrations remained stable in the uninfected control group. A restored anellovirus load was seen approximately 10 weeks after SARS-CoV-2 infection. For five subjects, an in-time anellome analysis via Illumina sequencing could be performed. In three of the five HCWs, the anellome visibly changed during SARS-CoV-2 infection and returned to baseline in two of these cases. In conclusion, anellovirus loads in blood can temporarily decrease upon an acute secondary infection.

## 1. Introduction

Anelloviruses infect the vast majority of the human population and represent the largest portion of the human blood virome [1]. Infection with anelloviruses occurs early in life and can be lifelong [2,3,4]. Although many studies have tried to associate anellovirus infection with disease since its discovery in 1997 [5], no link has been found, and it is hypothesized that anelloviruses are part of the healthy human virome [6,7].

The *Anelloviridae* virus family is immensely diverse, and within this family, three genera that infect humans can be distinguished: torque teno viruses (TTVs; *alphatorquevirus*), torque teno mini viruses (TTMVs; *betatorquevirus*) and torque teno midi viruses (TTMDVs; *gammatorquevirus*) [6]. Anelloviruses have a circular single-stranded negative sense DNA genome of 2.0–3.9 kb [6]. Their genome contains several overlapping open reading frames (ORFs), of which the largest, called ORF1, encodes the viral capsid protein [8].

All anelloviruses infecting an individual contribute to their personal “anellome”, which is relatively stable over time [3]. Increased concentrations of anelloviruses are found in the blood of people with immune disorders [8,9,10,11,12], indicating an interplay between anelloviruses and the host’s immune defence. However, little is known about the exact interaction between host immunity and anelloviruses. It is known that anelloviruses elicit an antibody response [13,14,15]. Next to that, anelloviruses are affected by innate immunity through anellovirus genome editing by Apolipoprotein B mRNA editing enzymes, catalytic polypeptide-like super family 3 (APOBEC3s) [16,17,18,19]. APOBEC3 proteins are cytidine deaminases that are able to edit viral genomes via deamination of cytosine (C) to uracil (U) [20]. We have shown that C-to-U editing leads to the production of dead-end virions, carrying a genome that has lost its capacity to code for functional proteins [18]. Other direct evidence of immunity against anelloviruses, e.g., other components of the innate immunity or T-cell immunity, is yet to be discovered.

In solid organ transplantation, a careful fine-tuning of immunosuppressive treatment is important to either prevent allograft rejection due to insufficient immunosuppression or to prevent infections in the case of excessive immunosuppression. It has been postulated that the pre- and post-transplantation comparison of TTV concentrations in blood and the examination of the rise in TTVs in the first two years after transplantation can guide immunosuppressive treatment dosing [21,22]. In the same line of thinking, one would expect, in an immunocompetent situation, the concentrations of human anelloviruses to decrease when immunity is activated, for instance during an acute respiratory infection.

To study the effect of a secondary viral infection on the concentration of human anelloviruses in blood, we investigated the blood anellome during a severe acute respiratory syndrome coronavirus 2 (SARS-CoV-2) infection in a cohort of hospital health care workers (HCWs) from the Amsterdam UMC hospital. In this cohort, serum was sampled every 4 weeks for a period of 12 weeks during the first pandemic wave in the Netherlands in 2020, when no vaccines were available yet and all SARS-CoV-2 infections were first-time SARS-CoV-2-infections. Sampling coincided with the first lockdown in the Netherlands and thereby the chance of infection by other (respiratory) viruses in the HCWs was minimal. From this cohort, we selected cases that were infected with SARS-CoV-2 during the study period and a control group of uninfected HCW. TTV, TTMV and TTMDV concentrations were measured before and after SARS-CoV-2 infection and compared to the anellovirus dynamics of HCWs without such infection. In addition, the HCWs completed questionnaires addressing their COVID-19 symptoms at each study visit.

## 2. Materials and Methods

### 2.1. Study Design and Participants

This S3 study is a prospective cohort study of health care workers (HCWs) at Amsterdam UMC and was conducted between March 2020 and June 2020 to analyse the risk factors and incidence of a first SARS-CoV-2 infection [23]. Serum samples were collected every 4 weeks and samples from the first 3 months of the follow-up were used in this study (T0, 4 weeks, 8 weeks and 12 weeks). SARS-CoV-2 infection was defined as a self-reported positive nucleic acid amplification test (NAAT) result and/or presence of SARS-CoV-2-specific antibodies, as detected by measuring total Ig against S1-RBD (Receptor Binding Domain) using a commercially available Wantai enzyme-linked immunosorbent assay (ELISA) [24]. The Wantai ELISA has a specificity of 99.6% and sensitivity of 95.4% in mild or asymptomatic cases 14 days after the onset of illness. All individuals reported on symptoms (either no symptoms or minimal, mild, moderate or severe symptoms) at every timepoint using surveys. Nineteen participants of this S3 study were selected for the SARS-CoV-2-SC (SARS-CoV-2 SeroConverting during follow-up) group. The selection criteria for this group were (1) negative S1-RBD total-Ig Wantai ELISA at T0; (2) no positive PCR test at T0; (3) positive Wantai ELISA at 4 weeks; (4) no serologic markers of a recent human coronavirus (HCoV; OC43, HKU1, 229E and NL63) infection at T0 [25]. Next to these 19 cases, 27 control individuals were included (SARS-CoV-2-NEG group). The criteria for inclusion as control were (1) negative S1-RBD total-Ig Wantai ELISA at T0, 4 weeks, 8 weeks and 12 weeks; (2) no SARS-CoV-2-positive PCR during the entire three-month follow-up; (3) no serologic markers of recent HCoV infection at T0. All individuals participated voluntarily and provided written informed consent and the medical ethical review board of the Amsterdam University Medical Centers approved the study (NL 73478.029.20, Netherlands Trial Register NL8645).

### 2.2. Nucleic Acid Isolation

The serum samples were stored at −80 °C. After thawing, 110 µL of the serum was centrifuged for 10 min at 5000× *g* and 100 µL of the supernatant was transferred to a new tube. The supernatant was treated with TURBO^TM^ DNase (12 µL DNase Buffer and 10 µL DNase, ThermoFisher, Karlsuhe, Germany, AM2238) to remove genomic and other microbial DNA [3]. DNase treatment has no positive or negative effect on anellovirus detection, but it is performed to remove naked background DNA from the host as it can compete in the Illumina sequencing. Nucleic acids were isolated according to the Boom isolation method [26] and then eluted in 65 µL of Baker water (VWR, 4218). Follow-up samples from each participant were in the same nucleic acid isolation run to ensure identical conditions during nucleic acid extraction. The nucleic acids were stored at −80 °C until further use.

### 2.3. Genera-Specific Quantitative PCRs (qPCRs)

Genera-specific qPCRs were performed using primers and probes as described [3]. The first qPCR detects *Alphatorqueviruses,* the second qPCR detects *Betatorquevirus* and the third qPCR detects *Gammatorquevirus*. Of note, there is some cross-reactivity by the TTMDV qPCR with TTMVs, and the qPCR detecting TTMDV can therefore be considered to detect TTMV + TTMDV. However, for clarity, this qPCR will be referred to as TTMDV qPCR throughout the manuscript. The qPCR reactions contained a 2.5 µL nucleic acid sample and 10 µL qPCR mastermix (6.25 µL 2x Qiagen Rotor-Gene QuantiNova probe pcr mastermix (Qiagen GmbH, Hilden, Germany, 208252), 0.25 µL genera-specific probe (10 µM), 0.25 µL genera-specific forward and reverse primer (both 20 µM) and 3 µL Baker water (VWR, 4218)). The reaction was performed as follows: 3 min hold on 95 °C followed by 40 cycles of 95 °C for 5 s and 60 °C for 5 s. The results were analysed using Rotor-Gene software (version 2.1.0). The detection limit was 10 DNA copies/reaction. Positive controls with known concentrations of the target-containing plasmid were used to calculate anellovirus DNA copies per mL serum. All samples were tested in duplicate and samples from each participant were in the same Rotor-Gene run to ensure identical qPCR conditions for all samples from each participant.

### 2.4. Illumina Next-Generation Sequencing Library Preparation for Anellome Analysis

Rolling circle amplification (RCA) was performed as previously described [3]. RCA-amplified nucleic acids were used as input to create a DNA library for Illumina NGS. The follow-up samples from each participant were processed in the same Illumina library preparation run and also analysed on the same Illumina MiSeq run, as described [3].

### 2.5. SCANellome—Prevalence of Anelloviruses

Raw Illumina reads (fastq format) were trimmed using Trimmomatic (version 0.39) on GITbash (version v2.35.2) and quality-checked using FastQC [3]. The trimmed reads were aligned to the SCANellome database (version 19 April 2023), which is an anellovirus database that contains 3636 human anellovirus ORF1 reference genomes (408 TTV, 1855 TTMV, 1373 TTMDV) [27]. Alignments were performed using Bowtie2 (version 2.2.5) with the “very sensitive” setting and the end-to-end mode on the Snellius cluster computer (https://www.surf.nl/). The aligned reads were sorted using Samtools (version 1.9) to generate a read to lineage table and a coverage per lineage table. Together, these tables were used to display the abundance of lineages with a genome coverage of >75%.

### 2.6. Variant Calling

APOBEC3 editing was detected by calculating variants on the anellovirus alignments using Lofreq (version 2.1.5), as previously described [18].

### 2.7. Luminex Assay

Antibody IgG titers against the Spike, Receptor Binding Domain and Nucleocapsid protein of wild-type SARS-CoV-2 were obtained using a Luminex MagPlex^®^ bead assay, as described [28].

### 2.8. Statistics

To test differences in sex and anellovirus incidence between the SARS-CoV-2-seroconverted and SARS-CoV-2-negative group, Fisher’s exact test was used. Differences in age and anellovirus load at T0 were tested using the Mann–Whitney U test. The association between anellovirus load at T0 and SARS-CoV-2 incidence was tested using logistic regression. We used univariable linear mixed regression analysis with a random intercept to compare changes in anellovirus load within subjects between the SARS-CoV-2-seroconverted and -seronegative groups. The data were log transformed before logistic and linear mixed regression analyses. Spearman correlation analyses were performed to correlate anellovirus load and SARS-CoV-2 antibody titer fold change between T0 and 4 weeks. Univariable linear regression and correlation analysis were performed in R (version 4.1.2). Graphpad (version 9.5.1) was used for additional data analysis and visualization. *p*-value was considered statistically significant at *p* ≤ 0.05.

## 3. Results

### 3.1. Descriptives

Forty-six health care workers (HCWs) were monitored every four weeks during the first COVID-19 wave in the Netherlands (March 2020–June 2020) (Table 1). Between the first visit (T0) and the visit at four weeks, 19 HCWs seroconverted for SARS-CoV-2 (the SARS-CoV-2-SC group). None of the participants in the SARS-CoV-2-SC group reported severe COVID-19 disease requiring hospital admission. Asymptomatic SARS-CoV-2-infection was reported by 7 (36.8%) participants, and 12 (63.2%) reported symptoms varying from minimal to moderate (Table 1). Symptom onset was on average 11 days after the start of this study. A control group was included with a total of 27 participants who remained SARS-CoV-2 PCR-negative and SARS-CoV-2-seronegative over the course of the follow-up (the SARS-CoV-2-NEG group). The two groups were comparable in age (*p* = 0.569, using Mann–Whitney U test) and sex (*p* = 0.716, using Fisher’s exact test).

Anellovirus loads (DNA copies per mL serum) were measured using TTV, TTMV and TTMDV qPCRs. At T0, out of the 19 SARS-CoV-2-SC participants, 8 tested positive (42.1%) for any anellovirus genus, and 16 out of the 27 SARS-CoV-2-NEG participants (59.3%) tested positive for anelloviruses (Table 1). There was no significant difference between anellovirus incidence (*p* = 0.370, using Fisher’s exact test; Table 1) and anellovirus load (*p* = 0.812, using Mann–Whitney U test) at T0 between the two groups. We also found no significant association between anellovirus load at T0 and the incidence of SARS-CoV-2 infection (*p* = 0.398, OR = 0.7553 [95% CI 0.3732 to 1.433], using simple logistic regression with likelihood ratio test; Table S1).

### 3.2. Short-Term Decrease in Anellovirus Load after SARS-CoV-2 Seroconversion

**Figure 1:**
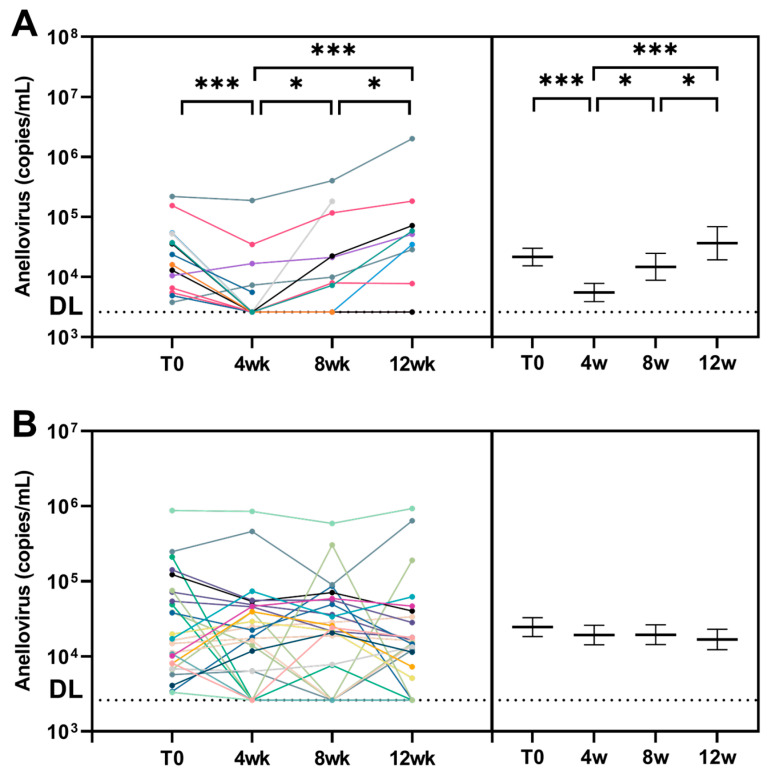
Anellovirus load in blood following SARS-CoV-2 infection. Anellovirus load (copies/mL), either TTV, TTMV or TTMDV, was determined in health care workers (HCWs) followed for three months (T0-12 weeks). The anellovirus load of each individual HCW (left) and mean with standard deviation (right) is shown. The anellovirus load was log transformed. Each color of the lines represents one health care worker (HCW). (**A**) SARS-CoV-2 seroconverters group. (**B**) SARS-CoV-2 negative group. Dashed line corresponds to the detection limit (DL). * = *p* ≤ 0.05, *** = *p* ≤ 0.001.

The dynamics of anellovirus load were determined in the two groups and compared over time using univariable linear mixed model regression. In the SARS-CoV-2-SC group, a significant decrease in anellovirus load was detected between T0 and 4 weeks (fold change (FC) of 0.256 [95% CI 0.125 to 0.525]; *p* < 0.001) (Figure 1A; Table S2). After 8 weeks, the anellovirus load increased and returned to baseline at 12 weeks (FC of 1.568 [95% CI 0.702 to 3.511]; *p* = 0.284). In the SARS-CoV-2-NEG group, anellovirus load remained stable during the three-month follow-up (Figure 1B; Table S2). Next, anellovirus load dynamics from baseline to 4 weeks were compared between the SARS-CoV-2-SC and SARS-CoV-2-NEG group, and the anellovirus load in the SARS-CoV-2-SC group was significantly decreased compared to the SARS-CoV-2-NEG group (FC of 0.327 [95% CI 0.135 to 0.796]; *p* = 0.017).

The three anellovirus genera were also considered separately. Here, the SARS-CoV-2-SC group showed a significant decrease in TTV load between T0 and 4 weeks (FC of 0.249 [95% CI 0.103 to 0.602]; *p* = 0.004) (Figure S1A; Table S2). TTV load returned to baseline at 12 weeks (FC of 2.604 [95% CI 0.965 to 7.092]; *p* = 0.074). In the SARS-CoV-2-NEG group, TTV load remained constant (Figure S1B; Table S2). TTV concentrations from baseline to 4 weeks were also compared between the SARS-CoV-2-SC and SARS-CoV-2-NEG group and a significant decrease was found (FC of 0.271 [95% CI 0.093 to 0.792]; *p* = 0.025). No statistical analysis could be performed for TTMV and TTMDV due to the low number of positives at T0 (Figure S1C–F).

**Figure 2:**
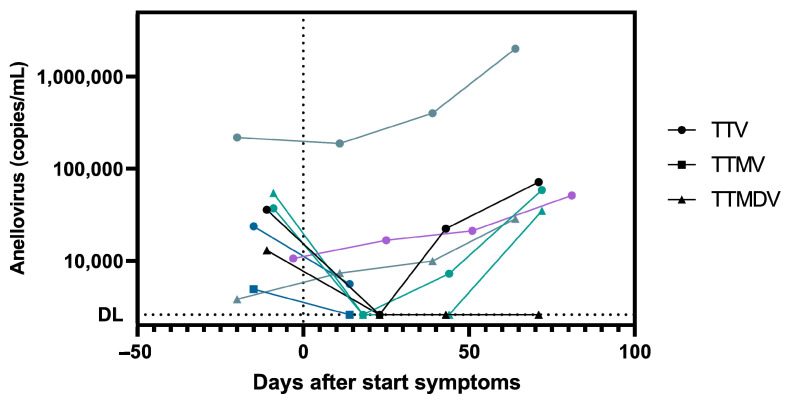
Anellovirus load after symptom initiation of SARS-CoV-2 infection. Anellovirus load (copies/mL) was determined in the SARS-CoV-2 seroconverter group during a follow-up of three months. Severity of symptoms and date of symptom onset were collected using surveys. Colors represent unique HCWs and symbols represent anellovirus genera: TTV (circle), TTMV (square) and TTMDV (triangle). Dashed line corresponds to the detection limit (DL).

At each study visit, individuals filled out surveys concerning their COVID-19 symptoms. Out of the 19 individuals of the SARS-CoV-2-SC group, 12 reported COVID-19 symptoms (Table 1). Given the survey information, we were able to compare the start of symptoms with anellovirus dynamics. In Figure 2, anellovirus concentrations relative to symptom onset are shown. A steep anellovirus decrease was observed 18 days after the start of symptoms. There were two odd ones out (S3-13 [TTV] and S3-15 [TTMDV]) in whom anellovirus concentrations increased after symptom onset.

**Figure 3:**
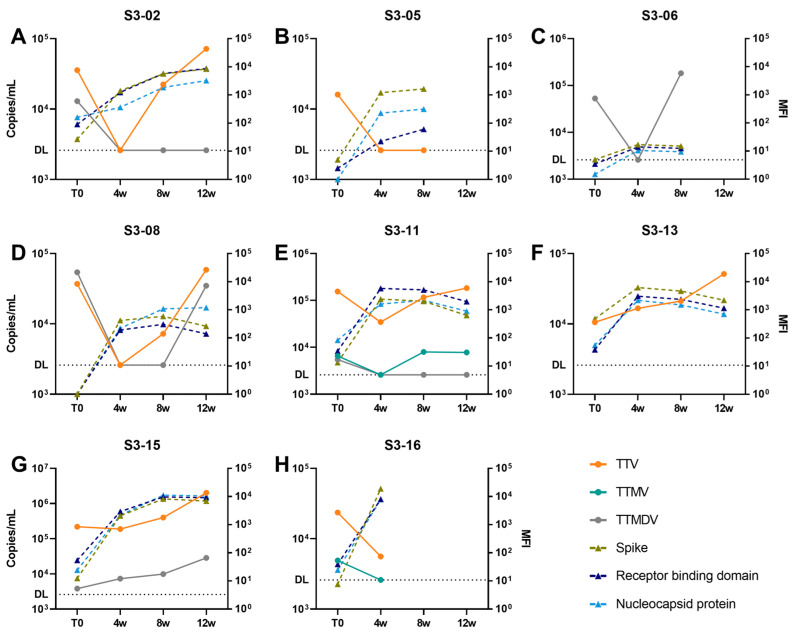
Anellovirus concentration and SARS-CoV-2 antibodies in blood following SARS-CoV-2 infection. (**A**–**H**). Anellovirus load (copies/mL; left Y-axis), either TTV (orange), TTMV (green) or TTMDV (grey), was determined in health care workers (HCWs—S3-02, S3-05, S3-06, S3-08, S3-11, S3-13, S3-15 and S3-16) followed for three months (T0-12 weeks). Antibodies targeting Spike (light green dotted line), Receptor Binding Domain (dark blue dotted line), and Nucleocapsid proteins (light blue dotted line) of SARS-CoV-2 are shown as median fluorescent intensity (MFI) on the right Y-axis. Dashed line corresponds to the qPCR detection limit (DL).

By definition, all individuals in the SARS-CoV-2-SC group seroconverted for antibodies recognizing proteins of SARS-CoV-2, but there was a large variation in quantitative antibody response following infection (Figure 3). To study the relation between quantitative antibody response and anellovirus load at 4 weeks, we looked at the rises of RBD-, S-, and N-antibody between T0 and 12 weeks. A significant relationship between the peak antibody median fluorescent intensity (MFI) and the dynamics of anelloviruses was found (Spike: R = 0.55, *p* = 0.042; RBD: R = 0.58, *p* = 0.028; and Nucleocapsid: R = 0.64, *p* = 0.014, using Spearman correlation, Figure S2), indicating that the steepest declines in anelloviruses between T0 and 4 weeks were associated with lower antibody response against SARS-CoV-2 proteins.

### 3.3. Anellome Alteration after SARS-CoV-2 Seroconversion

**Figure 4:**
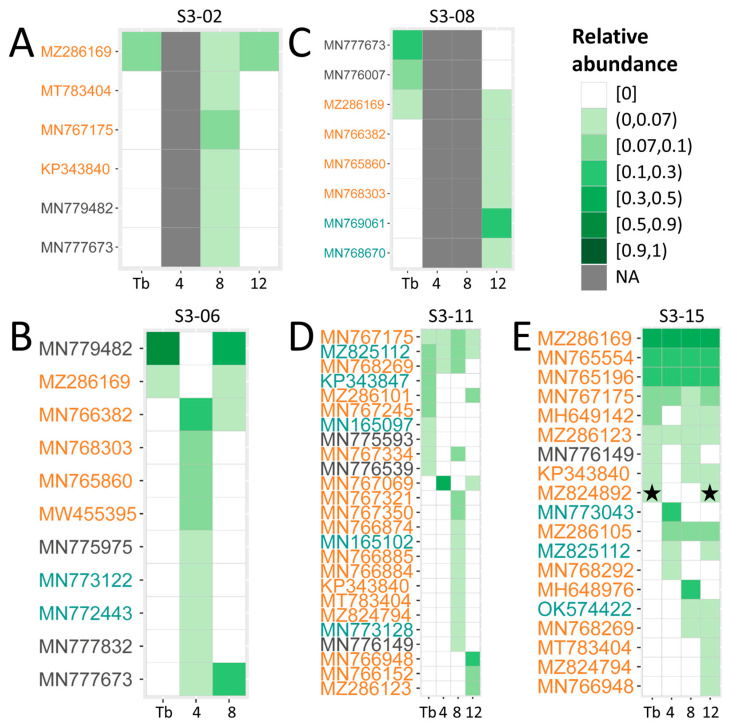
Blood anellome of individuals following SARS-CoV-2 infection. Heat maps of various anellovirus lineages and their abundance in health care workers (HCWs) followed for three months (T0, 4 weeks, 8 weeks and 12 weeks). The intensity of the green color refers to the relative abundance and the white color represents absence of a lineage in the sample. Dark grey squares indicate the sample was not included for next-generation sequencing due to absence of anelloviruses measured by qPCR. The colors of the reference genomes refer to different genera: TTV (orange), TTMV (cyan) and TTMDV (grey). (**A**) Individual S3-02, (**B**) individual S3-06, (**C**) individual S3-08, (**D**) individual S3-11, (**E**) individual S3-15. * indicates APOBEC3 editing.

Anellome dynamics following SARS-CoV-2 seroconversion were analyzed by charting anellomes using Illumina sequencing. Sera from the SARS-CoV-2-SC group that were positive for anelloviruses at T0 were subjected to rolling circle amplification (RCA; Table S3). Selection for sequencing was based on the presence of samples at at least three timepoints in the follow-up and a detectable anellovirus load after RCA at either 4 weeks, 8 weeks or 12 weeks. For five HCWs (S3-02, S3-06, S3-08, S3-11 and S3-15), a longitudinal anellome analysis could be performed. Sequencing resulted in an average of 3.77 × 10^6^ paired reads per sample (range 2.5 × 10^6^ to 4.42 × 10^6^) (Table S4). The reads were mapped to the SCANellome database (version 19 April 2023), a database containing the ORF1 reference sequences of 3636 representatives of human-infecting anelloviruses [27]. We used a cutoff of 75% read coverage against a SCANellome representative to determine lineage abundance. Per sample, a mean of 1.11 × 10^5^ anellovirus reads per million (RPM) were obtained, ranging between 2.84 × 10^1^ RPM for individual S3-02 (T0) and 4.29 × 10^5^ RPM for individual S3-15 (12 weeks). We next analyzed anellovirus richness (number of lineages found in one sample), based on categorizing via SCANellome representatives. Anellovirus richness varied, with individuals S3-02, S3-08 and S3-06 presenting the lowest richness, and individuals S3-11 and S3-15 the highest (Figure 4), which matches with their anellovirus concentrations (Figure 3). The number of anellovirus lineages at the start of the follow-up ranged from 1 (S3-02) to 10 lineages (S3-11) (Table S4).

Participant S3-02 started with one lineage at T0 (Figure 4A), a TTV, which matches with the virus detected by qPCR at T0 (Figure 3A). The TTMDV lineages of S3-02, supposedly present based on the TTMDV-qPCR at T0, were however not detected in the SCANellome analysis. At 4 weeks, thus after SARS-CoV-2 seroconversion, the anelloviruses of S3-02 were too low in concentration (all qPCRs negative) and therefore no anellome analysis could be performed. At 8 weeks, the S3-02 anellome contained five lineages which were not present or visible before SARS-CoV-2 infection; yet at 12 weeks, the original anellome from before SARS-CoV-2 infection was restored. The same phenomenon was seen for S3-06 (Figure 4B), where at 4 weeks the anellome was fully different from the T0 anellome, but restoration was seen at 8 weeks. The changing anellome of S3-06 is supported by the TTMDV qPCR data (Figure 3C). In participant S3-08, a changed anellome was observed after SARS-CoV-2 infection, yet the anellome was not restored to its T0 original form (Figure 4C). The anellome data do not fully match with the qPCR data, as we see that 12-week TTMDV lineages were not detected by TTMDV-qPCR (Figure 3D). In S3-11 (Figure 4D) and S3-15 (Figure 4E), lineages were observed that were present at T0 and not temporarily lost during SARS-CoV-2 infection. In addition, there are several extra lineages appearing and disappearing at 4 weeks, 8 weeks and 12 weeks.

We hypothesized that the lowering (and changing) of anelloviruses in blood may be caused by indirect APOBEC3 upregulation as part of the innate immune response during SARS-CoV-2 infection. APOBEC3 editing can be visible among Illumina reads, represented by at random C-to-U mutations in the genome, leading to the disruption of open reading frame of the *ORF1* gene and a dead end of the virus particle [18]. The Illumina reads were thus inspected for APOBEC3 editing. APOBEC3 editing was found in reads aligning with representative MZ824892 of individual S3-15 at two timepoints: T0 and 12 weeks (Table S5). An editing percentage, the number of C’s edited into U’s, of 25.8% and 38.9% was found at T0 and 12 weeks, respectively (Table S6; Figure 4). No APOBEC3 editing was found in any anellovirus lineage at 4 weeks and 8 weeks of S3-15, nor was it found in any of the anellovirus lineages of S3-02, S3-06, S3-08 or S3-11.

## 4. Discussion

In this study, we investigated human anelloviruses before and after SARS-CoV-2 infection. We showed that SARS-CoV-2 infection leads to a decrease in anellovirus load. This decrease was short-lived, as concentrations of anelloviruses, as well as the anellome, returned to baseline one or two months post SARS-CoV-2 infection.

Not only were we able to compare anellovirus dynamics longitudinally, starting prior to the infection, but we could also measure and compare anellovirus dynamics with uninfected controls of the same HCW cohort collected in the same calendar period. Next to that, we can assume that, due to lockdown measures in this calendar period, no other (respiratory) infections occurred in our study groups. Thus far, only a small number of studies have compared anellovirus concentrations in the context of a SARS-CoV-2 infection. Spezia et al. (2022) compared TTV load in SARS-CoV-2 PCR positive saliva of hospitalized COVID-19 patients and healthy subjects [29]. That study, however, found no difference in TTV load between the two groups, but it could be that the sampling in this study was too early in the SARS-CoV-2 infection. Thijssen et al. (2023) analyzed anellovirus load in plasma of hospitalized COVID-19 patients and they detected a significant anellovirus concentration drop from the day of hospitalization to day 6 after hospitalization [30]. Interestingly, a stronger decline in anellovirus load was found in COVID-19 patients with a hospital stay of fewer than 6 days compared to patients that stayed more than 6 days. Mendes-Correa et al. (2021) performed pre-emptive testing on individuals with SARS-CoV-2 infection-like symptoms and followed individuals who tested positive in time [31]. A reduction in TTV load in saliva was found 10 days after symptom onset, and the concentration dropped below detection in 10 out of 12 (83.3%) of their study subjects. We detected an anellovirus drop below our PCR detection limit in three out of seven (42.86%) TTV positives and PCR-undetectable anellovirus concentrations were on average found 18 days after symptom onset.

We found a significant correlation between antibody MFI targeting SARS-CoV-2 and a reduction in anellovirus load, meaning that the greater the decline in anellovirus concentration, the lower the production of SARS-CoV-2 antibodies. We expected the opposite relationship, as we assumed that high antibody production may be related to a strong immune activation and thus a strong decrease in anellovirus concentrations. However, it could be that anelloviruses are mostly cleared by the innate immune response and that a high innate immune activity is able to clear SARS-CoV-2 more rapidly compared to low innate immunity. High innate responses then result in fewer SARS-CoV-2-targeted antibodies.

In this cohort study, we selected all S3 study subjects who tested negative for Wantai-RBD-antibodies against SARS-CoV-2 at baseline (T0) while positive at the second timepoint. For one of our subjects, the Wantai ELISA test did not match with the Luminex-antibody tests that were also performed. Subject S3-13 had a notable Luminex signal for antibodies targeting the spike at the beginning of the study, whereas Wantai screening for RBD antibodies was below the cutoff. In this subject, the start of symptoms was very close to the start of this study, only three days after the first blood draw at T0, and it is therefore very likely that this person was, at T0, already infected by SARS-CoV-2. Interestingly, exactly this study subject (S3-13) is one of the two odd ones out in whom anellovirus load showed no decrease but an increase in the first 4 weeks. This observation strengthens the importance of the innate response in controlling anellovirus load very early after SARS-CoV-2 infection.

We were able to determine the anellome over time for five individuals. The anellome did not fully match the anellovirus load measured using genus-based qPCRs, and this was especially seen for individuals S3-02 and S3-08. Anelloviruses are extremely diverse and individuals can be infected with many lineages [3]. Even though the SCANellome database we used as reference contains 3636 different human anellovirus references [27], it is possible that not all lineages present in our individuals have a representative in the SCANellome database. On the other hand, the genus-specific PCR primers, designed on the most conserved region of the genome, may also have missed certain variants. Due to the huge diversity of anelloviruses, capturing all variations by universal PCRs is a challenge.

It is now well accepted that anellovirus levels increase upon immunosuppression [19,32,33,34,35,36,37]. Here, we show a decreasing concentration of anelloviruses upon SARS-CoV-2 infection. It is very unlikely that the coronavirus itself or SARS-CoV-2 antibodies caused a decrease in anellovirus concentrations. More plausible is the explanation that innate immune activation during acute infection has an effect on anelloviruses. When a virus infects a subject, the first line of defense is innate immunity, with the excretion of cytokines and interleukins by infected respiratory epithelial cells. One of these cytokines is interferon-α (IFN). It has been shown that TTV levels generally decrease in blood after IFN-α treatment in hepatitis C virus (HCV)-infected subjects [38,39,40,41]. Another important cytokine is IL-6, a molecule which is profoundly raised in severe COVID-19, but also considerably raised in non-severe COVID-19 [42]. IL-6 can act at a distance, and its increased levels are found in blood. It has been shown that both IFN-α and IL-6 can lead to increased expression of APOBEC3 in target cells [43,44,45,46], and if such increased APOBEC3-activity occurs in the cells where anelloviruses reside, deamination of cytosine in the genome of anelloviruses will lead to dead-end virions. We investigated whether we could detect APOBEC3 editing in the reads of the anellome shortly after SARS-CoV-2 infection; however, we saw no C-to-U editing in the sample where anellovirus concentrations were the most decreased. It could be that APOBEC3 editing was not observed because the dead-end virions were already removed from the circulation at 4 weeks and our sampling was too far apart. An alternative explanation for the decrease in anelloviruses coinciding with SARS-CoV-2 infection may lie in the host cells that produce anelloviruses. It has been postulated that T-cells form the main pool of cells producing anelloviruses [47]. COVID-19 patients, even mild cases, present with a temperate decrease in circulating T-cells (CD3^+^CD19^−^) [42], and thus SARS-CoV-2 infection may indirectly lead to decreasing anellovirus concentration in blood.

Our study participants were naïve to SARS-CoV-2 at the start of the study, as sampling occurred at the beginning of the pandemic (March 2020) and no vaccine was available yet. It would be interesting to investigate if an anellome is also impaired in a non-naïve situation, i.e., during a second infection by SARS-CoV-2 or a SARS-CoV-2 infection after having received a vaccination for the virus, or infections by other viruses, such as influenza virus. Maggi et al. (2005) investigated the TTV load in plasma and peripheral blood mononuclear cells of healthy adult individuals following hepatitis B virus vaccination—a new antigenic exposure—or influenza vaccination, which is a recall antigen, but in either case found no change in total TTV load at day 3, 7, 15, 30, 45, 60 or 90 post vaccination [48]. It could be that the innate immune response induced by vaccination is different from an actual infection, as no actively infected cells are involved.

Here, we show that anellovirus load decreases upon immune activation. It has been proposed that TTV levels can be used as a biomarker for immune status, especially to predict the success of immunosuppressive therapy in solid organ transplantation. A too high TTV load is associated with risk of infections, whereas a too low load is associated with the risk of organ rejection [21]. Clinical trials have now started to predict the success of organ transplantation in kidney and lung transplant patients using TTV load measurements [49,50]. A careful pre-transplantation TTV load determination, in the immunocompetent situation, is essential in this approach. However, here, we show that secondary infections may have an impact on TTV load in pre-transplantation testing and may thus deserve consideration.

## Figures and Tables

**Table 1:** Demographics table of SARS-CoV-2 seroconverters and SARS-CoV-2 negative controls.

	SARS-CoV-2 Seroconverters	SARS-CoV-2 Negatives	*p*-Value
Total number of subjects	19	27	-
Median age (IQR)	29 (25–45)	30 (25–55)	0.569 ^a^
Number of male participants (%)	3 (16)	6 (22)	0.716 ^b^
Days between T0 and symptom onset	11	NA	-
Severity of SARS-CoV-2 infection (%)			
No symptoms	7 (37)	NA	-
Minimal	5 (26)	NA	-
Mild	4 (21)	NA	-
Moderate	3 (16)	NA	-
SARS-CoV-2 seropositivity ^c^ (%)			
T0	0/19 (0)	0/27 (0)	-
4 weeks	19/19 (100)	0/27 (0)	-
8 weeks	17/17 (100)	0/27 (0)	-
12 weeks	14/14 (100)	0/27 (0)	-
Median SARS-CoV-2 antibody OD ^c^ (IQR)			
T0	0.05 (0–0.13)	0.03 (0–0.17)	-
4 weeks	12.05 (9.12–14.62)	0.01 (−0.01–0.02)	-
8 weeks	15.34 (7.21–18.25)	0.02 (0–0.04)	-
12 weeks	16.49 (14.11–18.19)	0.01 (−0.01–0.02)	-
SARS-CoV-2 PCR positivity (%)			
T0	0/5 (0)	0/4 (0)	-
4 weeks	8/8 (100)	0/2 (0)	-
8 weeks	NA	0/1 (0)	-
12 weeks	NA	0/1 (0)	-
Anellovirus positivity at T0 (%)			
Any anellovirus	8/19 (42)	16/27 (59)	0.370 ^b^
TTV + TTMV + TTMDV	1/19 (5)	3/27 (11)	0.632 ^b^
TTV ^d^	7/19 (37)	15/27 (56)	0.245 ^b^
TTMV ^d^	2/19 (11)	7/27 (26)	0.270 ^b^
TTMDV ^d^	5/19 (26)	4/27 (15)	0.456 ^b^
Median anellovirus load (DNA copies/mL) at T0 (IQR)			
All anelloviruses	19,849 (6290–53,222)	16,713 (7728–72,463)	0.812 ^a^
TTV	35,732 (16,080–155,034)	17,150 (8073–122,623)	0.407 ^a^
TTMV	5733 (4904–6562)	6655 (3410–54,093)	0.667 ^a^
TTMDV	12,974 (4641–53,573)	43,615 (21,830–65,848)	0.423 ^a^

IQR; interquartile range, OR; odds ratio, CI; confidence interval. ^a^ Mann–Whitney U test; ^b^ Fisher’s exact test; ^c^ Wantai ELISA; ^d^ at least positive for that specific genus.

## Data Availability

Host-removed reads can be found under NCBI Bioproject number PRJNA1046703 (RSA SRR26992979-SRR26992994). Titles in the reads refer to T1 = T0, T2 = 4 weeks, T3 = 8 weeks, T4 = 12 weeks.

## Supplementary Materials

The following supporting information can be downloaded at: https://www.mdpi.com/article/10.3390/v16010099/s1, Figure S1: Anellovirus genus load in blood following SARS-CoV-2 infection; Figure S2: Correlation plots of anellovirus load and SARS-CoV-2 antibody titer fold change; Table S1: Association between anellovirus load at T0 and SARS-CoV-2 incidence; Table S2: Univariable linear mixed model analysis of anellovirus load; Table S3: Anellovirus load after RCA in serum samples of individuals tested positive for SARS-CoV-2 infection; Table S4: Illumina results of SARS-CoV-2 positive individuals; Table S5: Variants of lineage MZ824892 infecting individual S3-15 at T0 and 12 weeks; Table S6: Variants of lineage MZ824892 infecting individual S3-15 at T0 and 12 weeks.
