# Supplementary material for: The Impact of First-Time SARS-CoV-2 Infection on Human Anelloviruses
Source: Viruses. 2024 Jan 9;16(1):99. doi: 10.3390/v16010099 (PMC10818381; doi:10.3390/v16010099)
Supplement: Supplementary file 1 [file viruses-16-00099-s001.zip › Supplementary Figures and Tables.pdf]

Supplementary Figures and Tables

The impact of first-time SARS-CoV-2 infection on human anelloviruses

Anne L. Timmerman [1,2], Lisanne Commandeur [1,2], Martin Deijs [1,2], Maarten G. J. M. Burggraaff [1,2], A. H. Ayesha Lavell [2,3], Karlijn van der Straten [1,2,4], Khadija Tejjani [1,2], Jacqueline van Rijswijk [1,2], Marit J. van Gils [1,2], Jonne J. Sikkens [2,3], Marije K. Bomers [2,3] and Lia van der Hoek [1,2,*]

[1] Laboratory of Experimental Virology, Department of Medical Microbiology and Infection Prevention, Amsterdam UMC, Location University of Amsterdam, Meibergdreef 9, 1105 AZ Amsterdam, The Netherlands
[2] Amsterdam Institute for Infection and Immunity, Meibergdreef 9, 1105 AZ Amsterdam, The Netherlands
[3] Department of Internal Medicine, Amsterdam UMC, Location Vrije Universiteit Amsterdam, De Boelelaan 1117, 1081 HV Amsterdam, The Netherlands
[4] Department of Internal Medicine, Amsterdam UMC, Location University of Amsterdam, Meibergdreef 9, 1105 AZ Amsterdam, The Netherlands
* Correspondence: c.m.vanderhoek@amsterdamumc.nl

Supplementary Figure S1, S2
Supplementary Table S1-S4 and S6

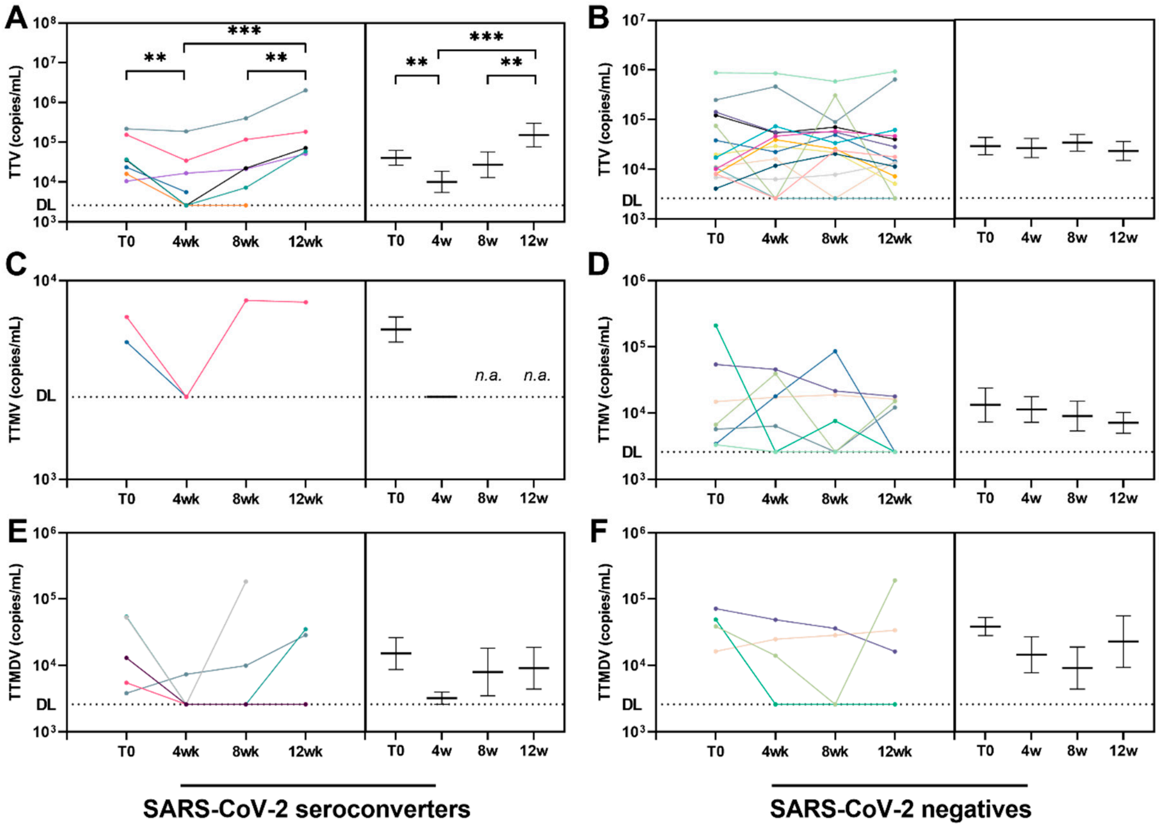

Figure S1. Anellovirus genus load in blood following SARS-CoV-2 infection. The anellovirus load (copies/mL), TTV (**a**+**b**), TTMV (**c**+**d**) and TTMDV (**e**+**f**), was determined in health care workers (HCWs) followed for three months (T0 – 12 weeks). The anellovirus load of each individual HCW(left), and mean with standard deviation (right) was shown. The anellovirus load was log transformed. Colors represent unique HCW. (**a**+**c**+**e**) SARS-CoV-2 seroconverters group. (**b**+**d**+**f**) SARS-CoV-2 negative group. Dashed line corresponds to the detection limit (DL).

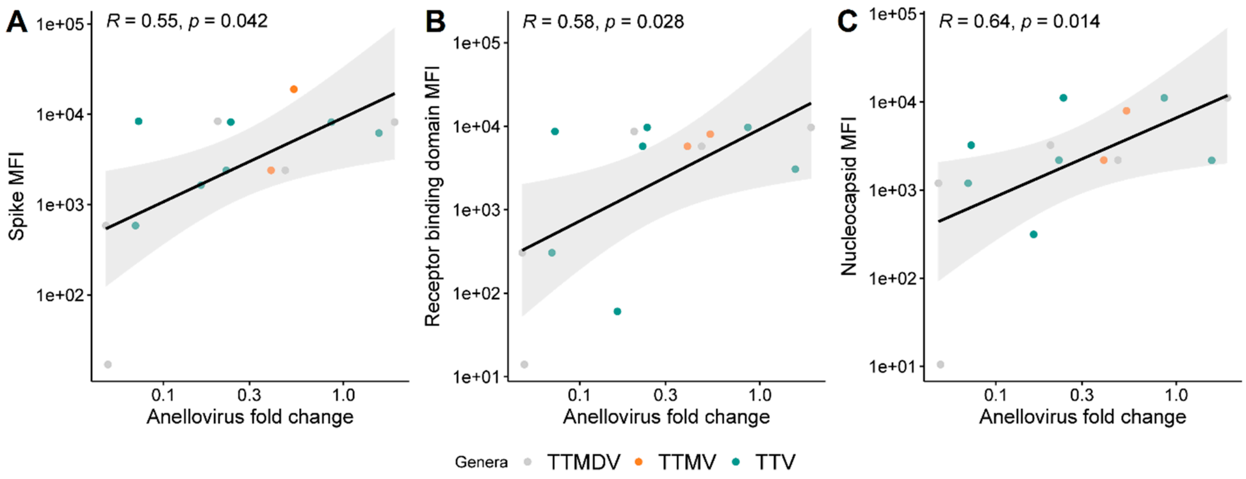

Figure S2. Correlation plots of anellovirus fold change and SARS-CoV-2 antibody MFI. The anellovirus concentration and antibodies against SARS-CoV-2 were determined in the SARS-CoV-2 seroconverters group. The anellovirus fold change was calculated between 4 weeks and T0 and antibody MFI represents highest value detected in the 3 month follow-up. Antibodies against the Spike (**a**), Receptor Binding Domain (**b**), and Nucleocapsid (**c**) of SARS-CoV-2 were measured. Black line and grey area represents the trend line and confidence interval (95%), respectively. The colors of the dots represent anellovirus genera (grey: TTMDV, orange: TTMV and blue: TTV).

Table S1. Association between anellovirus load at T0 and SARS-CoV-2 incidence. OR; odds ratio, CI; confidence interval.

	**P-value**	**OR (95% CI)**
**All anelloviruses**	0,398	0,755 (95% CI 0,373 to 1,433)
**TTV**	0,489	0,734 (95% CI 0,282 to 1,739)
**TTMV**	0,107	0,116 (95% CI 0,001 to 1,388)
**TTMDV**	0,829	1,165 (95% CI 0,270 to 4,760)

Table S2. Univariable linear mixed model analysis of anellovirus load. Geometric mean (95% CI), significance, and mean fold change (95% CI) of anellovirus load. SC = SARS-CoV-2 seroconverted group, NEG= SARS-CoV-2 negative group. CI = confidence interval.

Virus	Comparison 1, GM (95% CI)	Comparison 2, GM (95% CI)	P-value	Significance	Mean fold change (95% CI)
TTV	T0 (SC), 40579 (12708 to 129573)	4 weeks (SC), 10104 (4180 to 24429)	0,004	**	0,249 (0,103 to 0,602)
		8 weeks (SC), 25281 (9942 to 64480)	0,342	ns	0,623 (0,245 to 1,589)
		12 weeks (SC), 105749 (39199 to 285230)	0,074	ns	2,606 (0,966 to 7,029)
	4 weeks (SC)	8 weeks (SC)	0,068	ns	2,504 (0,984 to 6,385)
		12 weeks (SC)	0,000	***	10,486 (3,881 to 28,474)
	8 weeks (SC)	12 weeks (SC)	0,010	**	4,183 (1,519 to 11,577)
	T0 (NEG), 28854 (13056 to 63767)	4 weeks (NEG), 26459 (14456 to 48388)	0,786	ns	0,917 (0,501 to 1,677)
		8 weeks (NEG), 33903 (18524 to 62007)	0,616	ns	1,175 (0,642 to 2,149)
		12 weeks (NEG), 22997 (12580 to 42098)	0,481	ns	0,797 (0,436 to 1,459)
	4 weeks (NEG)	8 weeks (NEG)	0,443	ns	1,281 (0,697 to 2,356)
		12 weeks (NEG)	0,666	ns	0,87 (0,473 to 1,6)
	8 weeks (NEG)	12 weeks (NEG)	0,233	ns	0,679 (0,369 to 1,247)
	T0 (NEG)	4 weeks (SC)	0,025	*	0,271 (0,093 to 0,792)
ALL	T0 (SC), 21504 (9710 to 47619)	4 weeks (SC), 5505 (2688 to 11290)	0,000	***	0,256 (0,125 to 0,525)
		8 weeks (SC), 13784 (6473 to 29417)	0,262	ns	0,641 (0,301 to 1,368)
		12 weeks (SC), 33718 (15096 to 75501)	0,284	ns	1,568 (0,702 to 3,511)
	4 weeks (SC)	8 weeks (SC)	0,021	*	2,502 (1,175 to 5,333)
		12 weeks (SC)	0,000	***	6,117 (2,737 to 13,695)
	8 weeks (SC)	12 weeks (SC)	0,039	*	2,445 (1,076 to 5,562)
	T0 (NEG), 24465 (13657 to 43871)	4 weeks (NEG), 19181 (11327 to 32441)	0,375	ns	0,784 (0,463 to 1,326)
		8 weeks (NEG), 19352 (11450 to 32759)	0,394	ns	0,791 (0,468 to 1,339)
		12 weeks (NEG), 16759 (9908 to 28330)	0,168	ns	0,685 (0,405 to 1,158)
	4 weeks (NEG)	8 weeks (NEG)	0,973	ns	1,009 (0,596 to 1,709)
		12 weeks (NEG)	0,621	ns	0,874 (0,516 to 1,478)
	8 weeks (NEG)	12 weeks (NEG)	0,598	ns	0,865 (0,511 to 1,465)
	T0 (NEG)	4 weeks (SC)	0,017	*	0,327 (0,135 to 0,796)

Table S3. Anellovirus load after RCA in serum samples of individuals tested positive for SARS-CoV-2 infection. RCA; rolling circle amplification.

Serodate	Serodate	TTV (copies/mL)	TTMV (copies/mL)	TTMDV (copies/mL)
**S3-02**	**T0**	312917	2600	2600
**S3-02**	**4 weeks**	2600	2600	2600
**S3-02**	**8 weeks**	13978801324	2600	2600
**S3-02**	**12 weeks**	25093989902	2600	2600
**S3-06**	**T0**	2600	2600	3276573310
**S3-06**	**4 weeks**	2889370	7047	11853698843
**S3-06**	**8 weeks**	92964	15205	55514351777
**S3-08**	**T0**	77237533541	231456167	26502641513
**S3-08**	**4 weeks**	2600	2600	2600
**S3-08**	**8 weeks**	2600	2600	2600
**S3-08**	**12 weeks**	40618527	1092979844	9062855570
**S3-11**	**T0**	17414952917	133326597	19027197435
**S3-11**	**4 weeks**	31020567155	574511	2600
**S3-11**	**8 weeks**	30491016166	2049431619	339138238
**S3-11**	**12 weeks**	39918850957	44832122	2600
**S3-15**	**T0**	155728075848	217242987	8837610481
**S3-15**	**4 weeks**	70842290505	12826295291	5566940577
**S3-15**	**8 weeks**	201926938450	9375951065	10149647789
**S3-15**	**12 weeks**	635414067556	3531485194	13074209408

Table S4. Illumina results of the SARS-CoV-2 positive individuals. Reads and number of lineages present in the anellomes. Trimmed illumina reads were aligned to SCANellome anellovirus database (Laubscher et al., 2023). A coverage of 75% was considered a hit.

sample	Serodate	Illumina output			Number of TTV lineages	Number of TTMV lineages	Number of TTMDV lineages	Total number of anellovirus lineages
		Total reads	Anellovirus reads	Anellovirus reads per million (rpm)				
**S3-02**	**T0**	2922528	83	28	1	0	0	1
**S3-02**	**8 weeks**	4090494	134507	32883	4	0	2	6
**S3-02**	**12 weeks**	3830600	179158	46770	1	0	0	1
**S3-06**	**T0**	2504924	91564	36554	1	0	1	2
**S3-06**	**4 weeks**	3759680	697773	185594	4	2	3	9
**S3-06**	**8 weeks**	4095328	55320	13508	2	0	2	4
**S3-08**	**T0**	3633222	171482	47198	1	0	2	3
**S3-08**	**12 weeks**	3424138	9150	2672	4	2	0	6
**S3-11**	**T0**	4169126	105234	25241	5	3	2	10
**S3-11**	**4 weeks**	3715018	81631	21973	3	1	0	4
**S3-11**	**8 weeks**	3260626	812607	249218	11	3	1	15
**S3-11**	**12 weeks**	4266356	600818	140827	6	1	0	7
**S3-15**	**T0**	3829454	765920	200008	8	0	1	9
**S3-15**	**4 weeks**	4142204	520831	125738	7	2	0	9
**S3-15**	**8 weeks**	4417964	959427	217165	10	1	1	12
**S3-15**	**12 weeks**	4274382	1832059	428614	13	2	0	15

Table S5: See Excel file

Table S6. Variants table of lineage MZ824892 infecting individual S3-15 in T0 and 12 weeks. Trimmed illumina reads were aligned to SCANellome anellovirus database (Laubscher et al., 2023). A coverage of 75% was considered a hit. Variants were called using Lofreq by using the positive strand (Timmerman *et al.,* 2022*)*

**Serodate**	**Percentage of total Cs edited**
T0	26
12 weeks	39
